# Association between plasma neurofilament light chain (NfL) concentrations and renal impairment

**DOI:** 10.1002/alz.085240

**Published:** 2025-01-09

**Authors:** Susan Ashrafzadeh‐Kian, Bethany Larson, Joshua A Bornhorst, Alicia Algeciras‐Schimnich

**Affiliations:** ^1^ Mayo Clinic, Rochester, MN USA

## Abstract

**Background:**

Neurofilament Light Chain (NfL) is a blood biomarker of axonal injury and neurodegeneration that can be used in a variety of neurological disorders. Despite the potential clinical use of plasma NfL across multiple neurological disorders, there is increasing evidence that underlying comorbidities such as renal impairment associated with chronic kidney disease (CKD) and cardiovascular diseases can increase NfL concentrations. The objective of this study was to determine the relationship between plasma NfL concentrations and renal function (CKD staging) in individuals without known neurological conditions.

**Methods:**

Individuals (n=62) seen in an outpatient setting at Mayo Clinic who had various stages of renal impairment (stages 1‐5 by CKD‐EPI 2021 estimated glomerular filtration rate (eGFR) equation) and did not have co‐existing conditions that are known to elevate plasma NfL were retrospectively identified through electronic medical record review. EDTA plasma from these patients collected within +/‐ 30 days of the eGFR determination was identified and stored frozen (‐80°C) until the time of testing. Samples were tested using the Simoa® NF‐light™ V2 Advantage Kit (Quanterix). NfL concentrations in pg/mL were used to calculate age‐adjusted Z‐scores (https://shiny.dkfbasel.ch/baselnflreference/) using an online calculator. Correlation analysis was performed to assess associations of continuous eGFR (mL/min/ 1.73 m^2^) with plasma NfL age‐adjusted Z‐scores.

**Results:**

A strong negative correlation between NfL Z‐score and eGFR was observed (Spearman’s ρ= ‐0.806). Mean NfL Z‐scores (ranges) in each CKD stage were ‐0.4(‐2.3‐1.4) for stage 1 (N=9), 0.9(‐0.8‐1.9) for stage 2 (N=10), 1.9(‐1.2‐3.4) for stage 3 (N=29), 2.9(2.7‐3.5) for stage 4 (N=6), and 3.2(1.6‐3.7) for stage 5 (N=8); with all groups showing significant differences (P>0.05) except between groups 4 and 5 (P=0.09).

**Conclusions:**

Plasma NfL concentrations were increased in individuals with a decreased eGFR. Kidney impairment is an important potential confounding factor to consider when utilizing NfL as a biomarker of neurodegeneration in the clinical setting.

